# Myocarditis Mimicking Takotsubo Cardiomyopathy With First Dose of Neoadjuvant Nivolumab–Relatlimab

**DOI:** 10.1155/crom/1443004

**Published:** 2025-12-10

**Authors:** Rohit Rao, Felicia Tejawinata, Tejasi Sachdeva, Imran Rashid, Michael Zacharias, Jessica Siegel, McKay Herpel, Akihiro Yoshida, Luke D. Rothermel, Iris Y. Sheng, Ankit Mangla

**Affiliations:** ^1^ Department of Hematology and Oncology, University Hospitals Seidman Cancer Center, Cleveland, Ohio, USA; ^2^ Department of Hematology and Oncology, Case Western Reserve University School of Medicine, Cleveland, Ohio, USA, case.edu; ^3^ Department of Radiology, University Hospitals Cleveland Medical Center, Cleveland, Ohio, USA, uhhospitals.org; ^4^ Department of Cardiology, University Hospitals Cleveland Medical Center, Cleveland, Ohio, USA, uhhospitals.org; ^5^ Department of Pathology, University Hospitals Cleveland Medical Center, Cleveland, Ohio, USA, uhhospitals.org; ^6^ Department of Pharmacy, University Hospitals Seidman Cancer Center, Cleveland, Ohio, USA; ^7^ Department of Dermatology, Case Western Reserve University School of Medicine, Cleveland, Ohio, USA, case.edu; ^8^ Department of Surgical Oncology, University Hospitals Cleveland Medical Center, Cleveland, Ohio, USA, uhhospitals.org; ^9^ Department of Developmental Therapeutics, Case Comprehensive Cancer Center, Cleveland, Ohio, USA

**Keywords:** anti-LAG3, anti-PD1, melanoma, myocarditis, neoadjuvant immunotherapy, nivolumab–relatlimab, Takotsubo cardiomyopathy

## Abstract

Neoadjuvant use of immune checkpoint inhibitors (ICIs) is the new standard of care in patients with clinical stage III melanoma. However, it is associated with immune‐related adverse events (irAEs). Nivolumab–relatlimab in the neoadjuvant setting is an NCCN‐recommended treatment for patients with clinical stage III melanoma. Anti‐LAG3 molecule comes with an increased risk of cardiac irAE, especially myocarditis. Takotsubo cardiomyopathy (TTC), a reversible decline in heart function driven by catecholamine overload, is reported as a cardiac irAE in the literature. However, the mechanism of TTC being an irAE is elusive. It is known that myocarditis and TTC share a lot of common features, although the presence of cardiac inflammation essentially rules out TTC. Here, we report the case of an elderly patient with a history of heart failure with midrange ejection fraction, diagnosed with clinical stage III melanoma, who developed shortness of breath with the first dose of neoadjuvant nivolumab–relatlimab. Cardiac magnetic resonance (CMR) imaging demonstrated a severe apical hypokinesis and no myocardial edema, suggestive of TTC. However, since myocarditis could not be ruled out, the patient was started on high‐dose methylprednisolone followed by a 9‐week taper of prednisone. The CMR changes reverted to baseline 44 days later, with the patient experiencing complete recovery. He underwent wide local excision of the primary melanoma and complete lymph node dissection, which showed a major pathologic response. Postoperatively, he remains on surveillance with no evidence of recurrence. This report emphasizes early recognition of cardiac irAEs and initiation of corticosteroids, which could help prevent morbid long‐term complications.

## 1. Introduction

Neoadjuvant use of immune checkpoint inhibitors (ICIs) is the new standard of care for patients presenting with clinical stage III melanoma (CS3M) [1]. Two randomized control trials, the S1801 (using pembrolizumab monotherapy) and the NADINA trial (using ipilimumab and nivolumab dual checkpoint inhibitors), showed a significantly better event‐free survival (EFS) in patients with CS3M receiving immunotherapy [2, 3]. The combination of nivolumab (antiprogrammed cell death protein‐1 [anti‐PD1]) and relatlimab (antilymphocyte activation gene‐3 [anti‐LAG3]) is approved for patients with metastatic or locally advanced unresectable melanoma [4]. A single‐arm, Phase II trial exploring the neoadjuvant use of nivolumab–relatlimab (Nivo‐Rela) showed a significantly high number of patients with CS3M achieving pathologic complete response (pCR) [5]. The three regimens are recommended by the NCCN (National Comprehensive Cancer Network) in the neoadjuvant setting for patients with CS3M. Although all ICIs are associated with cardiac immune‐related adverse events (irAEs), anti‐LAG3 medications are particularly associated with a higher incidence of cardiac events [6, 7].

Takotsubo cardiomyopathy (TTC) is a transient, nonischemic cardiomyopathy thought to arise secondary to emotional stress and catecholamine overload [8]. Although TTC is reported as an irAE, no mechanistic relationship has been established explaining the causal effect of ICIs in TTC [9]. On the other hand, myocarditis is a rare yet established irAE of ICIs [10]. The incidence of myocarditis with the use of antilymphocyte antigen 3 (LAG3) agents is higher than previously thought [6]. Here, we report the case of an elderly man with a history of nonischemic cardiomyopathy and heart failure with midrange ejection fraction (HFmrEF), who developed myocarditis mimicking TTC‐like features after the first dose of neoadjuvant Nivo‐Rela for CS3M. This report examines the mechanistic relationship between cardiac inflammation and the antiprogrammed death‐1 (anti‐PD1) axis, emphasizing the importance of prompt recognition of myocarditis to prevent fatality and facilitate recovery.

## 2. Case Report

### 2.1. History of Presentation

A 78‐year‐old man presented to surgical oncology with a diagnosis of 1.4‐cm deep, nonulcerated melanoma of the back. Clinical exam was positive for a palpable lymph node in the left axilla, which was biopsied and found positive for melanoma. Staging PET/CT (Positron‐Emission Tomography/Computed Tomography) scan and MRI brain showed that the lymph node was the only FDG (Fluorodeoxyglucose)‐avid spot with no other evidence of metastasis (Figure 1a). The patient met with the medical oncologist to discuss neoadjuvant immunotherapy. He signed consent for Nivo‐Rela and received the first dose within a week of completing the staging workup. Four weeks following the first dose, he presented to the emergency room (ER) with an acute onset of nonexertional, nonpositional, constant left lower chest pain without dyspnea or palpitations for 1 day. His vitals were stable at presentation.

Figure 1(a) Pretreatment PET/CT showing FDG‐avid lymph node, (b) repeat PET/CT scan 6 weeks after Cycle 1, (c) repeat PET scan 7 months after Cycle 1, and (d) STIR imaging of heart showing pericardial but not myocardial inflammation.

**Figure:**
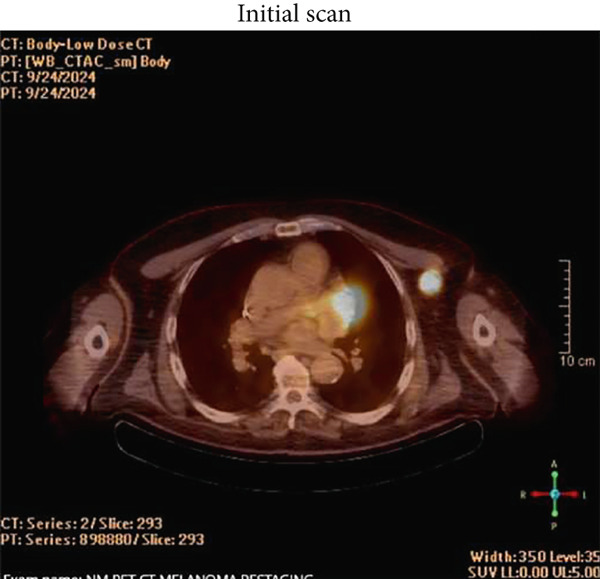
(a)

**Figure:**
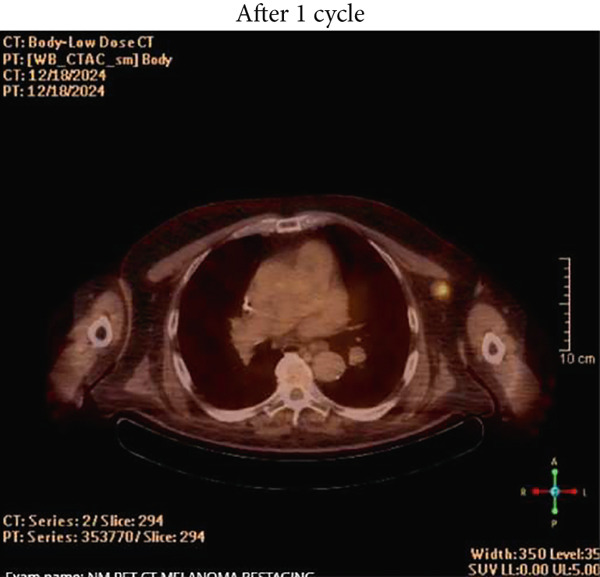
(b)

**Figure:**
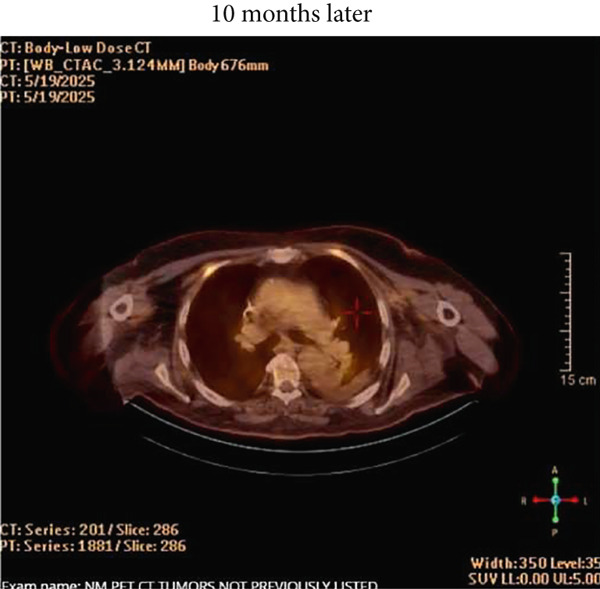
(c)

**Figure:**
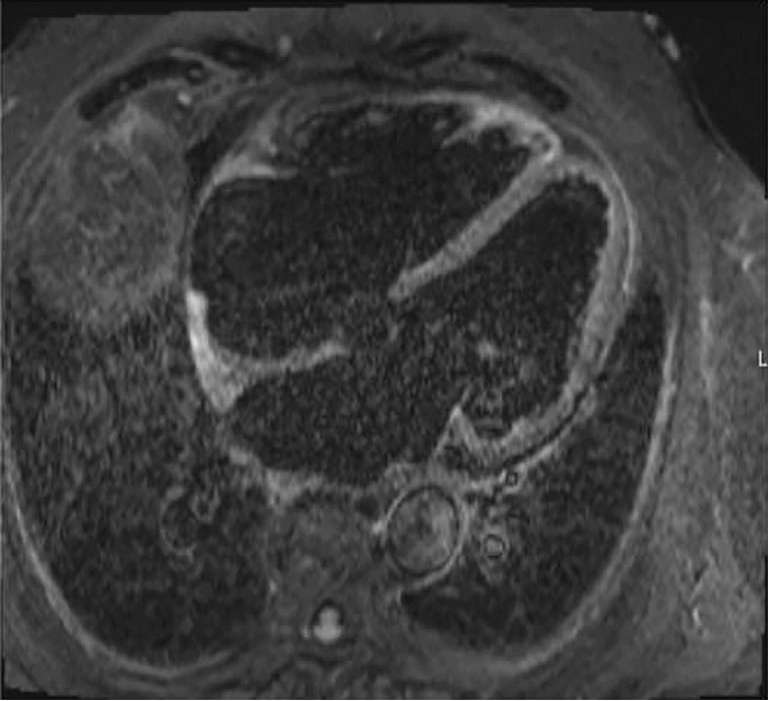
(d)

### 2.2. Past Medical history

The patient′s medical history includes chronic kidney disease (stage IIIa), nonischemic cardiomyopathy and HFmrEF, ascending aortic aneurysm, paroxysmal atrial flutter, first‐degree atrioventricular (AV) block, Mobitz Type I secondary AV block, right bundle branch and left anterior fascicular block, and pacemaker placement in January 2024. Prior to the initiation of Nivo‐Rela, the ejection fraction on a two‐dimensional transthoracic echocardiogram (TTE) was estimated at 40%–45%, which had remained stable for several years. His last cardiac catheterization, 10 months prior to presentation, was significant for 40% stenosis in the distal‐left anterior descending artery and no significant stenosis in the right, left, or circumflex coronary artery. He started on neoadjuvant Nivo‐Rela with a plan for complete lymph node dissection (CLND) after two cycles of immunotherapy.

### 2.3. Differential Diagnosis

The presentation and the history of use of the ICI raised the suspicion of immunotherapy‐induced myocarditis, pericarditis, and myopericarditis. The extensive history of cardiac events raised the possibility of acute coronary syndrome and acute‐on‐chronic heart failure.

### 2.4. Investigations

The electrocardiogram (EKG) was significant for chronic T‐wave inversions and no ST‐segment changes. The initial reading for high‐sensitivity troponin was 271 ng/L, which trended to 412 ng/L by 10 h, stabilized at 429 ng/L by 24 h, and started trending down by 72 h. Creatine kinase MB level was 0% at presentation. Serial measurements of CK‐MB remained zero over the next 4 days. Chest X‐ray showed no pleural effusion or pulmonary vascular congestion. Initial TTE demonstrated global hypokinesis of the left ventricle (LV) with an EF of 40%–45%.

Cardiac MRI (CMR) performed on Day 3 of admission noted a dilated LV, EF of 40%, and severe hypokinesis of apical anterior/inferior/septal/lateral walls and true apex. STIR (Short Tau Inversion Recovery) imaging showed no myocardial enhancement (Figure 1d). T1‐ and T2‐weighted images could not be obtained due to the presence of an intracardiac device. The pericardium adjacent to the apex and apical lateral wall had increased signal on STIR imaging, suggesting pericardial inflammation. Endomyocardial biopsy was deferred due to the high risk of perforation.

### 2.5. Management

Due to concern for myocarditis/pericarditis, methylprednisolone dosed at 1 mg intravenously every 24 h was started in the ER. He was transitioned to prednisone 1 mg/kg and was discharged after 4 days with a 9‐week steroid taper. A myocardial biopsy was proposed for the patient to confirm the diagnosis of myocarditis. However, the patient refused the procedure, citing the high risk of perforation associated with the procedure.

### 2.6. Outcome and Follow‐Up

The patient finished the prednisone taper over the next 8 weeks without any incident or recurrence of his symptoms. Follow‐up CMR 44 days later showed resolution of previously noted wall motion abnormalities, normal STIR imaging, and uniformly nulled myocardium, suggesting that previous wall motion abnormalities were not ischemic in origin. The LV remained dilated with improvement in EF to 47% (baseline). PET/CT done 6 weeks after Cycle 1 of Nivo‐Rela showed a near‐complete radiologic/metabolic response (Figure 1b). Considering the Grade 4 cardiac irAE with the first dose of ICI, additional treatment with Nivo‐Rela was forgone. The patient underwent wide local excision of the skin lesion and CLND 3 months later. Major pathologic response (MpR) was noted in the index lymph node (less than 10% viable neoplasm), and pCR was noted in the skin lesion. Follow‐up PET/CT scans continue to show no radiologic evidence of recurrence (Figure 1c). A 2D echo done after 6 months showed an EF of 48% with moderate dilation in the left ventricular cavity size.

## 3. Discussion

Myocarditis is a fatal irAE from the use of checkpoint inhibitors, with up to 50% mortality [11]. Hence, prompt recognition and initiation of steroids are critical not only to prevent mortality but also to prevent long‐term sequelae. Myocarditis has a varied presentation ranging from chest pain with no other complications to life‐threatening arrhythmias and heart failure [12]. Myocardial biopsy is the gold standard for diagnosis; however, it is fraught with the danger of myocardial perforation. Over the last two decades, clinical practice relies on a combination of clinical presentation, lab tests, and imaging studies, particularly the cardiac MRI [12]. TTC, on the other hand, is a nonischemic cardiomyopathy associated with catecholamine surge, although the pathophysiology is not entirely clear [8]. Recent studies suggest a complex interplay between the PD‐1/PD‐L1 axis and cardiac myocytes in modulating inflammation in “physiologic” stress‐induced cardiomyopathy [13]. Isoproterenol (ISO), a synthetic beta‐adrenergic agonist, can induce a TTC‐like cardiomyopathy in mice [14]. A single intraperitoneal dose of ISO in the mouse induces an acute inflammatory response in the heart and a TTC‐like picture in the LV [13]. Single‐cell RNA sequencing showed that ISO‐mediated cardiac injury resulted in a transient increase in the expression of PD‐1 and PD‐L1 in the immune cells (including macrophages and dendritic cells) residing in the myocardium. After ISO‐injection, treatment with anti‐PDL1 antibody increased the inflammation in the myocardium [13]. In addition, ISO injection in a PD‐1 mouse prolonged the duration of inflammation and severity of LV dysfunction compared with wild type mice, indicating the role of the PD‐1/PD‐L1 axis in TTC [13]. ICI‐induced myocarditis is thought to be a T‐cell–mediated injury facilitated by ICI use [15].

Ruling out myocarditis is one of the quintessential features in diagnosing TTC, although the clinical and radiologic picture of TTC and myocarditis can overlap [16]. CMR is a valuable tool to differentiate; however, imaging features can overlap. In the correct clinical setting, the presence of either myocardial edema or nonischemic myocardial injury is sufficient to diagnose myocarditis [15]. Regional or global wall motion abnormalities and evidence of pericarditis can support the diagnosis of myocarditis in the absence of either of the major criteria. Interestingly, TTC can also present with similar findings on CMR, making it difficult to distinguish between the two [17]. A biopsy of the myocardium could differentiate between the two, but in our case, the patient refused to consent, citing the risk of perforation. Persistence of cardiac changes on CMR makes myocarditis more probable [17]. In our patient, the apical wall hypokinesis resolved on the follow‐up CMR. In the absence of a biopsy, it is challenging to determine if this was true TTC or a response to early initiation of high‐dose steroids in a patient with ICI‐induced myocarditis. The management of TTC is supportive care [18]. However, in the context of ICI use, where PD‐1/PD‐L1 blockade may worsen the intensity and duration of inflammation in the cardiac myocyte, early initiation of steroids may be of use. Of the 17 patients with TTC‐like cardiac irAE reviewed in the literature, nine received steroids, and eight of those nine patients recovered clinically [9]. From a cardiac standpoint, our patient recovered to his baseline cardiac function. Considering the Grade 4 cardiac irAE, we permanently discontinued the checkpoint inhibitor.

### 3.1. Conclusion

In the context of checkpoint inhibitor use, the acute phase of myocarditis may look like TTC. Although a myocardial biopsy is the gold standard for diagnosis, it may not be feasible due to the risk of perforation. The advances in understanding the role of the PD‐1/PD‐L1 axis in the pathophysiology of TTC indicate that early initiation of steroids, even in those suspected of TTC, may facilitate quicker clinical recovery.

## Consent

All the patients allowed personal data processing, and informed consent was obtained from all individual participants included in the study.

## Conflicts of Interest

The authors declare no conflicts of interest.

## Funding

No funding was received for this manuscript.

## Data Availability

The data that support the findings of this study are available from the corresponding author upon reasonable request.

## References

[1] Mangla A. , Lee C. , Mirsky M. M. , Wang M. , Rothermel L. D. , Hoehn R. , Bordeaux J. S. , Carroll B. T. , Theuner J. , Li S. , Fu P. , and Kirkwood J. M. , Neoadjuvant Dual Checkpoint Inhibitors vs Anti-PD1 Therapy in High-Risk Resectable Melanoma: A Pooled Analysis, JAMA Oncology. (2024) 10, no. 5, 612–620, 10.1001/jamaoncol.2023.7333, 38546551. 38546551 PMC10979364

[2] Patel S. P. , Othus M. , Chen Y. , Wright G. P. , Yost K. J. , Hyngstrom J. R. , Hu-Lieskovan S. , Lao C. D. , Fecher L. A. , Truong T.-G. , Eisenstein J. L. , Chandra S. , Sosman J. A. , Kendra K. L. , Wu R. C. , Devoe C. E. , Deutsch G. B. , Hegde A. , Khalil M. , Mangla A. , Reese A. M. , Ross M. I. , Poklepovic A. S. , Phan G. Q. , Onitilo A. A. , Yasar D. G. , Powers B. C. , Doolittle G. C. , In G. K. , Kokot N. , Gibney G. T. , Atkins M. B. , Shaheen M. , Warneke J. A. , Ikeguchi A. , Najera J. E. , Chmielowski B. , Crompton J. G. , Floyd J. D. , Hsueh E. , Margolin K. A. , Chow W. A. , Grossmann K. F. , Dietrich E. , Prieto V. G. , Lowe M. C. , Buchbinder E. I. , Kirkwood J. M. , Korde L. , Moon J. , Sharon E. , Sondak V. K. , and Ribas A. , Neoadjuvant–Adjuvant or Adjuvant-Only Pembrolizumab in Advanced Melanoma, New England Journal of Medicine. (2023) 388, no. 9, 813–823, 10.1056/NEJMoa2211437, 36856617. 36856617 PMC10410527

[3] Blank C. U. , Rozeman E. A. , Fanchi L. F. , Sikorska K. , van de Wiel B. , Kvistborg P. , Krijgsman O. , van den Braber M. , Philips D. , Broeks A. , van Thienen J. V. , Mallo H. A. , Adriaansz S. , ter Meulen S. , Pronk L. M. , Grijpink-Ongering L. G. , Bruining A. , Gittelman R. M. , Warren S. , van Tinteren H. , Peeper D. S. , Haanen J. B. A. G. , van Akkooi A. C. J. , and Schumacher T. N. , Neoadjuvant Versus Adjuvant Ipilimumab Plus Nivolumab in Macroscopic Stage III Melanoma, Nature Medicine. (2018) 24, no. 11, 1655–1661, 10.1038/s41591-018-0198-0, 2-s2.0-85054564616, 30297911. 30297911

[4] Paik J. , Nivolumab Plus Relatlimab: First Approval, Drugs. (2022) 82, no. 8, 925–931, 10.1007/s40265-022-01723-1, 35543970. 35543970

[5] Burton E. M. , Milton D. R. , Tetzlaff M. T. , Wani K. , Ross M. I. , Postow M. A. , Lazcano R. , Glitza I. C. , Wong M. K. , Patel S. P. , Diab A. , Gershenwald J. E. , McQuade J. L. , Warner A. B. , Prieto V. G. , Lee J. E. , Goepfert R. P. , Fisher S. B. , Song A. , Malke J. , Simon J. M. , Ariyan C. , Torres-Cabala C. A. , Davies M. A. , Lazar A. , Wargo J. A. , Tawbi H. A. , and Amaria R. N. , Long-Term Survival and Biomarker Analysis Evaluating Neoadjuvant Plus Adjuvant Relatlimab (Anti-LAG3) and Nivolumab (Anti-PD1) in Patients With Resectable Melanoma, Journal of Clinical Oncology. (2025) 43, no. 26, 2856–2862, 10.1200/JCO-25-00494, 40638872. 40638872 PMC12252214

[6] Gautam S. S. S. , Rajak K. , Matai P. , Ng W. L. , Martinez E. C. , and Atrash A. , Increasing Incidence Of Myocarditis Associated With Nivolumab/relatlimab (Opdualag), a Retrospective Analysis Of FDA Adverse Event Reporting System Database, Journal of Cardiac Failure. (2025) 31, no. 1, 10.1016/j.cardfail.2024.10.122.

[7] Moslehi J. , Lichtman A. H. , Sharpe A. H. , Galluzzi L. , and Kitsis R. N. , Immune Checkpoint Inhibitor–Associated Myocarditis: Manifestations and Mechanisms, Journal of Clinical Investigation. (2021) 131, no. 5, 10.1172/JCI145186, 33645548. PMC7919710 33645548

[8] Lyon A. R. , Citro R. , Schneider B. , Morel O. , Ghadri J. R. , Templin C. , and Omerovic E. , Pathophysiology of Takotsubo Syndrome: *JACC* State-of-the-Art Review, JACC. (2021) 77, no. 7, 902–921, 10.1016/j.jacc.2020.10.060. 33602474

[9] Trontzas I. P. , Vathiotis I. A. , Kyriakoulis K. G. , Sofianidi A. , Spyropoulou Z. , Charpidou A. , Kotteas E. A. , Syrigos K. N. , and ImmunoTTS Collaborative Group , Takotsubo Cardiomyopathy in Cancer Patients Treated With Immune Checkpoint Inhibitors: A Systematic Review and Meta-Summary of Included Cases, Cancers (Basel). (2023) 15, no. 9, 10.3390/cancers15092637, 37174104. PMC10177389 37174104

[10] Naqash A. R. , Moey M. Y. Y. , Tan X.-W. C. , Laharwal M. , Hill V. , Moka N. , Finnigan S. , Murray J. , Johnson D. B. , Moslehi J. J. , and Sharon E. , Major Adverse Cardiac Events With Immune Checkpoint Inhibitors: A Pooled Analysis of Trials Sponsored by the National Cancer Institute—Cancer Therapy Evaluation Program, Journal of Clinical Oncology. (2022) 40, no. 29, 3439–3452, 10.1200/JCO.22.00369, 35658474. 35658474 PMC10166352

[11] Ball S. , Ghosh R. K. , Wongsaengsak S. , Bandyopadhyay D. , Ghosh G. C. , Aronow W. S. , Fonarow G. C. , Lenihan D. J. , and Bhatt D. L. , Cardiovascular Toxicities of Immune Checkpoint Inhibitors, JACC. (2019) 74, no. 13, 1714–1727, 10.1016/j.jacc.2019.07.079, 2-s2.0-85072228965. 31558256

[12] Basso C. , Myocarditis, New England Journal of Medicine. (2022) 387, no. 16, 1488–1500, 10.1056/NEJMra2114478. 36260793

[13] Hayashi T. , Tiwary S. K. , Lavine K. J. , Acharya S. , Brent M. , Adamo L. , Kovacs A. , and Mann D. L. , The Programmed Death-1 Signaling Axis Modulates Inflammation and LV Structure/Function in a Stress-Induced Cardiomyopathy Model, JACC: Basic to Translational Science. (2022) 7, no. 11, 1120–1139, 10.1016/j.jacbts.2022.05.006, 36687266. 36687266 PMC9849278

[14] Liao X. , Chang E. , Tang X. , Watanabe I. , Zhang R. , Jeong H. W. , Adams R. H. , and Jain M. K. , Cardiac Macrophages Regulate Isoproterenol-Induced Takotsubo-Like Cardiomyopathy, JCI Insight. (2022) 7, no. 3, 10.1172/jci.insight.156236, 35132957. PMC8855841 35132957

[15] Palaskas N. , Lopez-Mattei J. , Durand J. B. , Iliescu C. , and Deswal A. , Immune Checkpoint Inhibitor Myocarditis: Pathophysiological Characteristics, Diagnosis, and Treatment, Journal of the American Heart Association. (2020) 9, no. 2, e013757, 10.1161/JAHA.119.013757, 31960755. 31960755 PMC7033840

[16] Ghadri J.-R. , Wittstein I. S. , Prasad A. , Sharkey S. , Dote K. , Akashi Y. J. , Cammann V. L. , Crea F. , Galiuto L. , Desmet W. , Yoshida T. , Manfredini R. , Eitel I. , Kosuge M. , Nef H. M. , Deshmukh A. , Lerman A. , Bossone E. , Citro R. , Ueyama T. , Corrado D. , Kurisu S. , Ruschitzka F. , Winchester D. , Lyon A. R. , Omerovic E. , Bax J. J. , Meimoun P. , Tarantini G. , Rihal C. , Y-Hassan S. , Migliore F. , Horowitz J. D. , Shimokawa H. , Lüscher T. F. , and Templin C. , International Expert Consensus Document on Takotsubo Syndrome (Part I): Clinical Characteristics, Diagnostic Criteria, and Pathophysiology, European Heart Journal. (2018) 39, no. 22, 2032–2046, 10.1093/eurheartj/ehy076, 2-s2.0-85048669355, 29850871. 29850871 PMC5991216

[17] Fernández-Pérez G. C. , Aguilar-Arjona J. A. , de la Fuente G. T. , Samartín M. , Ghioldi A. , Arias J. C. , and Sánchez-González J. , Takotsubo Cardiomyopathy: Assessment With Cardiac MRI, American Journal of Roentgenology. (2010) 195, no. 2, W139–W145, 10.2214/AJR.09.3369, 2-s2.0-77955633795. 20651173

[18] Singh T. , Khan H. , Gamble D. T. , Scally C. , Newby D. E. , and Dawson D. , Takotsubo Syndrome: Pathophysiology, Emerging Concepts, and Clinical Implications, Circulation. (2022) 145, no. 13, 1002–1019, 10.1161/CIRCULATIONAHA.121.055854, 35344411. 35344411 PMC7612566

